# Positron Annihilation Studies of Hydrostatically Extruded AA1050 Aluminum

**DOI:** 10.3390/ma18184428

**Published:** 2025-09-22

**Authors:** Ewa Dryzek, Mirosław Wróbel, Maciej Sarnek, Jacek Skiba

**Affiliations:** 1 Institute of Nuclear Physics, Polish Academy of Sciences, PL-31342 Kraków, Poland; 2 Faculty of Metals Engineering and Industrial Computer Science, AGH University of Krakow, Mickiewicza 30 Ave., 30-059 Kraków, Poland; mwrobel@agh.edu.pl; 3 Institute of Quality Sciences and Product Management, Cracow University of Economics, Rakowicka 27 Str., 31-510 Kraków, Poland; sarnekm@uek.krakow.pl; 4 Institute of High Pressure Physics Polish Academy of Sciences (Unipress), ul. Sokołowska 29, 01-142 Warszawa, Poland; jacek.skiba@unipress.waw.pl

**Keywords:** aluminum, hydrostatic extrusion, plastic deformation, recrystallization, mechanical properties, positron annihilation spectroscopy, variable energy positron beam, crystallographic texture

## Abstract

AA1050 aluminum was hydrostatically extruded at room temperature to true strains of 0.9 and 3.2, and at cryogenic temperature to a true strain of 0.9. As a result of the extrusion process, the yield strength (YS) increased by 130–160% to 120–130 MPa, and the ultimate tensile strength (UTS) rose by 64–81% to 125–140 MPa. The hardness reached 46–49 HV. YS and UTS values correspond to mechanical properties typical of the H6 or H8 temper designations, with unusually high elongation at break ranging from 15% to 16.4%. Differences in lattice parameters, crystallite size, and lattice strain between samples deformed under various conditions—as well as those annealed after deformation—were within the margin of measurement uncertainty. This indicated that differences in defect density between the samples were relatively small, due to dynamic recovery occurring during extrusion. However, positron annihilation spectroscopy demonstrated that the cryo-cooled material extruded at a true strain of 0.9, as well as the one extruded at RT at a true strain of 3.2, exhibited significantly higher mean lattice defect concentrations compared to the sample extruded at RT at a true strain of 0.9. The predominant defects detected were vacancies associated with dislocations. The extrusion parameters also significantly affected the crystallographic texture. In particular, they altered the relative proportions of the <111> and <100> components in the axial texture, with the <100> component becoming dominant in cryogenically extruded samples. This trend was further intensified during recrystallization, which enhanced the <100> component even more. Recrystallization of the deformed materials occurred in the temperature range of 520–570 K. The activation energy for grain boundary migration during recrystallization was estimated to be approximately 1.5 eV.

## 1. Introduction

The global aluminum extruded products market size was estimated to be worth USD 91 billion in 2024, with an estimated compound annual growth rate (CAGR) of 8.4% during 2025–2030 [1]. However, the share of the Al alloys from the 1xxx family (i.e., commercially pure aluminum with purity over 99 wt.%), which includes AA 1050 alloy, is relatively small because of their softness. For this reason, such alloys are seldom used for structural applications. However, their low density combined with excellent corrosion resistance and workability, high electrical and thermal conductivity, and highest weldability among aluminum alloys predisposes them to applications in electrotechnics, electronics, packaging, and kitchenware. Long products of these alloys (such as bars, wires, and profiles) are often shaped by a high-temperature extrusion (about 450–550 °C [2]) to reduce work hardening and to improve productivity and surface finish. This temperature corresponds to a homologous temperature of 0.77–0.78 and is higher than the pure metal homologous temperature of the recrystallization (typically about 0.4). Recrystallization as a diffusion-driven process depends on temperature and time. It can be limited to some extent by quenching the product with water after it has passed through the die. The reduction ratio and the exit speed up to 100 and up to 75 mm/s, respectively, are not uncommon (but less common when extruding profiles [3]) and depend on each other as well as on the available pressure and extrusion temperature. Therefore, an economically acceptable way to eliminate recovery and recrystallization is virtually impossible while maintaining high efficiency, i.e., maximizing the deformation rate. The operating window of extrusion parameters must be carefully selected from the limit diagrams, such as those described in Ref. [4] (Sections 4.13 and 5.2.1 and in Figure 5.23). In particular, heterogeneity in the flow rate and stress distribution over the extrusion product cross-section resulting from friction against the die causes product discontinuities and cracking, e.g., crow’s feet and fir-tree cracking, which can be formed at too high deformation rates.

The increase in the deformation temperature usually improves metal plasticity but also intensifies recovery and recrystallization processes. These processes should be minimized to ensure significant strain hardening and thus eliminate important disadvantages of the aluminum alloys from the 1xxx family. Therefore, forming should take place at most at room temperature (RT), which is possible at relatively low deformation rates. At economically acceptable deformation rates, it is also possible to reduce the heterogeneity of the extruded material flow rate and stress distribution over its cross-section, consequently preventing product discontinuity by appropriate die geometry and minimizing friction by lubrication. Near-perfect lubrication can be provided by the hydrostatic extrusion (HE) process, in which the billet is surrounded by pressurized liquid, i.e., there is no friction between the container walls and billet and friction of the die can be largely reduced by a film of pressurized lubricant between the extrusion product and die (when designing presses, it is assumed that the working liquid does not surround the die orifice). In such conditions, the shear stress on the die face is small and can be neglected, as was assumed by Pugh and Ashcroft [5]. Thus, the billet extrusion occurs under hydrostatic conditions formulated by Sachs et al. [6]

To the best of our knowledge, the HE process was first patented by James Robertson between 1891 and 1899 (a series of British and US patents [7,8,9,10,11,12,13,14,15]), but Kronberger [16] attributed its first implementation into practice to Percy Williams Bridgman, who was awarded the Nobel Prize in 1946 for his studies of materials at high temperatures and pressures. Indeed, in 1951, he received a US patent for this method and apparatus [17]. Since then, HE technology has been intensively researched in the USA, Europe, and Japan (a short overview of older studies can be found elsewhere, e.g., [4,18]). The improvement continued, and new patents related to this technology appeared, e.g., [19,20,21,22]. Any product suitable for extrusion by conventional technology can be extruded by HE, but the extrusion ratio and exit rate can reach the value of 10,000:1 [23] and 2.5 m/s [24], respectively. Hydrostatically extruded aluminum has been reported to have better mechanical properties than its cold-drawn counterparts [25]. A significant increase in the strength of HE aluminum was confirmed by, among others, Herø and Mikkelsen [26] and Lee et al. [27], and related to the subgrain refinement [28]. Hydrostatic extrusion results in a more uniform strain distribution across the cross-section and a more homogenous microstructure of the product. Hydrostatic compressive stresses increase ductility, allowing for greater deformation without failure, lowering the deformation temperature, and refining microstructure as was reported for aluminum [29,30]. Therefore, the product properties, such as increased strength, fatigue resistance, and corrosion resistance, are superior to those achieved through conventional plastic working methods, such as rolling, drawing, or hot extrusion [30,31,32,33,34,35,36,37].

The current work aims to investigate the effect of the HE temperature and strain rate on the mechanical properties, thermal stability, and recrystallization of the AA 1050 alloy. Its novelty lies in the combination of X-ray diffraction and positron annihilation techniques used in this type of material research. In particular, positron annihilation spectroscopy is known as a valuable non-destructive tool for studies of changes in the microstructure of metals and alloys due to its exceptional sensitivity to open-volume defects of the crystal lattice, such as vacancies, vacancy clusters, voids, and dislocations [38,39]. The presence of defects alters the annihilation characteristics, allowing for their identification and tracking of changes in their concentration. Unlike transmission electron microscopy (TEM) techniques, where significant recovery must be taken into account when preparing and observing pure aluminum samples, the methods we use enable the examination of bulk samples for which such effects can be negligible.

## 2. Materials and Methods

Commercial aluminum of 99.5% purity (AA1050) in the form of rods was deformed by the single-step HE according to the specifications in Table 1. Before extrusion, the billets were annealed at 623 K for 1 h to alleviate the effects of previous mechanical processing and to produce homogeneous coarse grains of ca. 30 μm. The billet extruded at low temperature (referred to as cryo-cooled) was immersed in a liquid nitrogen (LN) bath for 1 h immediately before extrusion. The time between removing the billet from the LN container and starting its extrusion did not exceed 15 s. Room temperature oil and propylene glycol overcooled to −30 °C were used as the pressure-transmitting medium for the RT and low temperature HE, respectively. The HE processing parameters were selected to ensure that the extrusion temperatures of the samples differed, despite having a similar reduction ratio (*R*) of approximately 2.5. Sample Al-2.5 was extruded at RT, and sample Al-2.5C was cryo-cooled before HE. The third sample (labeled Al-25) was extruded at RT at a reduction ratio ten times higher, i.e., 25.3.

One-step HE was performed at the Institute of High Pressure Physics (Unipress), Warsaw, Poland. The extrusion die with an opening angle 2α of 45° was used. Before starting the extrusion process, the die opening was closed with a charge cone that matched the die opening and apex angle. After loading the billet into the chamber, it was flooded with a high pressure medium, which was then compressed with a movable piston on one side, closing the working chamber. The pressure medium surrounded the entire not-encapsulated billet; thus, it did not come into contact with the chamber walls. The pressure of the pressure-transmitting medium was gradually increased to the critical value at which extrusion started. The samples were not cooled after exit. The product surface temperature was contact-measured using a Pt-PtRh thermocouple (Simex Ltd., Gdańsk, Poland) immediately after extrusion. It was equal to 262 K, 332 K, and 361 K for Al-2.5C, Al-2.5, and Al-25, respectively. The increase in the sample temperature due to almost adiabatic deformation was therefore ca. 37 K and 66 K for Al-2.5 and Al-25 material, respectively. Determining the temperature increase in sample Al-2.5C was not possible because its exact temperature before the start of the HE process was not known precisely.

Mechanical properties were determined by the tensile tests and Vickers hardness measurements (under a load of 0.2 kgf) performed following the recommendations of the relevant standards, i.e., [40,41,42,43]. The result of the tensile tests was considered correct if the crack occurred in the middle of the gage length measured by an extensometer. Hardness measurements were performed on the billets and the extruded rods’ cross-section.

Samples used for hardness, XRD, and for the positron annihilation measurements were ground using SiC papers (grids from 500 to 1200) and deeply etched in a Keller’s solution to remove a subsurface layer deformed by cutting and grinding.

The annealing regimes of the samples were as follows. For the positron annihilation measurements, the isochronal annealing from room RT up to 630 K with a step of 20 K in a nitrogen atmosphere was performed (20 min each step). After each annealing step, the positron annihilation spectroscopy measurements were carried out at RT. For X-ray measurements, the samples were annealed at 590 K for one hour. The positron annihilation and X-ray diffraction (XRD) measurements were done for both the as-deformed material and the material annealed after deformation. The reference sample was annealed at 773 K in a vacuum (~10^−4^ Pa) for 1 h and then slowly cooled inside the furnace.

A Panalytical Empyrean diffractometer (Malvern Panalytical, Almelo, The Netherlands) was used for the X-ray measurements. Ni-filtered Cu radiation (the filter thickness was 0.02 mm) was applied. A parallel Goebel mirror (length 55.3 mm, acceptance angle 0.8°) and Soller slit (length 0.04 rad) collimated the incident beam. The same type of Soller slit, together with the parallel plate collimator (opening 0.18°), was placed in front of the PIXcel 3D detector (Malvern Panalytical, Almelo, The Netherlands). X-ray patterns were recorded from the symmetric diffraction data (2 theta in the range of 20–150°, step 0.02°). The trial and error (TREOR) methodology developed by Werner et al. [44] and the Dicevol procedure developed by Boultif and Louër [45] were applied to determine the crystal structure and the lattice parameters. The methodology of Williamson and Hall [46] was used to determine the crystalline size and lattice strain. Calculations were performed using software HighScore Plus v.3.05 (Malvern Panalytical, Almelo, The Netherlands). Our previous study demonstrated that these parameters are sensitive to the recovery of deformed metal, and the crystallographic texture is more sensitive to recrystallization [47,48,49,50].

The crystallographic texture was calculated from the incomplete pole figures 111, 200, 220, and 311 measured on an equiangular measurement grid using the Schulz reflection methodology [51]. The range of the azimuthal angle (*α*) and the polar angle (*β*) were 0–80° and 0–360°, respectively. The angle step was Δ*α* = Δ*β* = 5°. The orientation distribution function (ODF) and complete pole figures were calculated from the incomplete pole figures for the *α* angle range of 0–60° using the discrete Arbitrarily Defined Cell (ADC) methodology [52] implemented in the LaboTex software v. 3.0 by LaboSoft [53]. According to Caleyo et al., this methodology reproduces the texture well throughout its entire sharpness range [54]. Lack of sample symmetry and the cubic crystal symmetry confirmed by XRD measurements (space group Fm-3m/No. 225 and Laue class O_h_) were confirmed and assumed in these calculations.

The variable energy positron (VEP) beam was used [55] to determine the positron diffusion length in the material studied. The positrons emitted from the ^22^Na source of 30 mCi activity were moderated using frozen neon, accelerated to an appropriate energy in the range of 50 eV to 30 keV, and formed into a monoenergetic beam of 5 mm diameter, guided to the sample. The beam intensity was 10^6^ e^+^/s. Annihilation quanta (511 keV) were registered by the Doppler broadening of the annihilation line spectrometer (Ametek Ortec, Oakridge, TN, USA) with the HpGe (1.2 keV FWHM energy resolution, interpolated at 511 keV). Based on the annihilation spectra, two commonly used parameters, *S* and *W*_r_, were determined. The *S* parameter is defined as the ratio of the area under the fixed central part of the annihilation line to the area under the whole annihilation line. It is sensitive to the annihilation of positrons with low-momentum electrons present in open volume defects. The *W*_r_ parameter is calculated as the ratio of the area under the fixed wing region of the annihilation line to the area under the whole annihilation line. It is influenced by the annihilation with high-momentum electrons, i.e., the core ones, and is sensitive to the chemical environment at the annihilation site.

For positron annihilation lifetime spectroscopy (PALS) measurements, the digital spectrometer manufactured by TechnoAP (Hitachinaka, Japan) with BaF_2_ scintillators connected to the H3378-50 photomultipliers (Hamamatsu Photonics, Iwata City, Japan) was employed. The time resolution of the spectrometer was about 210 ps (full width at half maximum). The positron source, consisting of a ^22^Na isotope with an activity of 40 μCi, enveloped in a 7 μm thick Kapton foil, was used in the measurements. The analysis of the spectra containing at least 10^6^ counts was performed by applying the LT code, subtracting background, and taking into account source contribution [56].

## 3. Results and Discussion

The selected HE process parameters, the properties of the initial material, and the extruded material are summarized in Table 1. The noted increase of 131–159% in YS, 64–81% increase in UTS, and 1.5 times greater hardness due to extrusion are not surprising. It is important that these increases were accompanied by an elongation at break of 15–16% Thus, YS and UTS reach the level typical of H6 or H8, treatments for which the elongation at break is only 6–7% and the hardness is no higher than 44 HV [57]. The improvement in mechanical properties is therefore significant. However, the differences in the HE process parameters for RT extrusion have a relatively weak impact on the mechanical properties of the product. This can be attributed to the relatively low sensitivity of the mechanical properties of technically pure Al to the strain rate in the range used in this work [58,59]. The extrusion temperature appears to exert a more substantial influence on the resultant material properties. For the cryo-cooled sample with true strain of 0.9, the YS and UTS are about 10% higher, and the elongation at break is about 8% lower than for the samples extruded at RT.

### 3.1. X-Ray Diffraction

The lattice parameters, crystallite size, and lattice strain of the samples deformed under different conditions, as well as samples annealed after deformation, are shown in Table 2. The differences between the samples practically fall within the measurement uncertainty, which can be related to comparatively small differences in the defect density due to dynamic recovery during extrusion as a result of the deformation temperature. Indeed, the homologous deformation temperature of HE is close to 0.3–0.4, i.e., it falls into the range typical for pure metals recrystallization. Moreover, the relatively high deformation rate of HE results in intensive dynamic recovery, which seems to be responsible for the relatively low difference in the mechanical properties and the microstructure parameters collected in Table 1 and Table 2. The results for the samples annealed at 590 K for 1 h indicate the stability of the lattice parameters, crystallite size, and lattice strain (Table 2). A tendency for a decrease in the lattice parameter due to annealing could be associated with static recovery and/or recrystallization. However, the differences between the samples before and after annealing seem to be very small.

**Figure 1:**
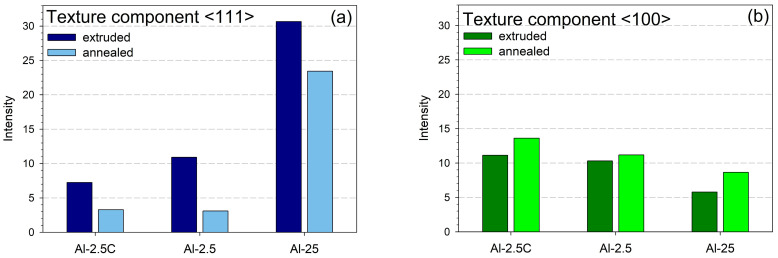
Intensity of the components of the axial textures: (**a**) <111>; (**b**) <100>.

Both the deformed and post-deformation annealed samples exhibited distinct axial textures typical of axisymmetric deformation, e.g., extrusion or drawing of face-centered cubic (fcc) metals. Particularly, good axial symmetry was visible in the deformed state. The relative contributions of the main components of the texture, i.e., <111> and <100>, depend on the extrusion conditions (Figure 1). For the most strongly deformed material, Al-25, the <111> component dominates over the <100> one, which is typical for highly deformed fcc metals with a high stacking fault energy (SFE), such as aluminum. The deformation textures of the remaining, much less deformed materials are more similar to those obtained for metals with lower SFE, strongly deformed in classical uniaxial deformation processes. For the Al-2.5 sample, the contributions of components <111> and <100> are nearly equal. For the Al-2.5C sample, the contribution of component <100> is greater than that of the <111> one. The samples Al-2.5 and Al-2.5C differ mainly in the deformation temperature.

Similar texture effects were previously registered for classical uniaxial deformation and related to the SFE value of fcc metals. A relationship between the relative intensity of the <100> and <111> components and the SFE in fcc metals was proposed by English et al. [60]. From Figure 2 in Ref. [60], one can conclude that an increase in the <100> component contribution in the texture of pure metal with fcc structure can be related to a decrease in SFE. However, large uncertainties in the temperature dependence of the SFE were noticed already by Hirth and Lothe [61]. Both increasing and decreasing tendencies of the SFE dependence on temperature could be found among the results of theoretical calculations [62,63,64,65,66,67]. For aluminum, a decreasing trend of the SFE with increasing temperature was reported in Refs. [68,69,70,71] However, between cryogenic temperature and RT, the effect is rather small. Nevertheless, the implementation of TEM or X-ray diffraction techniques for the SFE measurement poses serious challenges, especially for pure, low-melting-point metals with high SFE, such as aluminum. The use of TEM is easier for low SFE metals. In this case, an increase in SFE with increasing temperature was reported [65,66,72]. The same tendency was observed in X-ray diffraction measurements [73,74,75] and seems to also apply to the current results. However, the difference in the strain rate, which is higher for the cryo-extruded sample, also has some influence on SFE, and presumably this effect is stronger. Indeed, Hu et al. previously reported that an increase in the contribution of the <100> texture component can be related to a decrease in the deformation temperature and an increase in the strain rate (see Table 2 in Ref. [76]). There is experimental evidence that high strain rates and nanocrystalline microstructure may favor mechanical twinning in pure aluminum, e.g., Refs. [77,78], respectively, although this deformation process is typical for low SFE metals with fcc structure. Thus, it can be concluded that the texture effect observed for the cryo-cooled material can be related more to a high strain rate and a refined microstructure typical for hydrostatic extrusion processing at cryogenic temperatures. An increase in the intensity of the <100> texture component, as illustrated in Figure 1, combined with a decrease in the intensity of the <111> component due to annealing, can be related to recrystallization.

Our previous studies have shown that positron annihilation techniques are highly sensitive tools for studying recovery and recrystallization processes [47,50]. Thus, this technique was used to study the course of recrystallization in detail (Section 3.2).

### 3.2. The Positron Annihilation Studies

#### 3.2.1. Extruded Material

The VEP beam application allowed us to obtain the values of positron diffusion length, which, together with positron lifetime results, may provide information on the defect types and densities [79]. The analysis of the VEP measurement results requires knowledge of the positron implantation profile. This profile for monoenergetic positrons entering a semi-infinite material can be described using the Gaussian derivative, which can be expressed as the Makhovian function [80].

**Figure 2:**
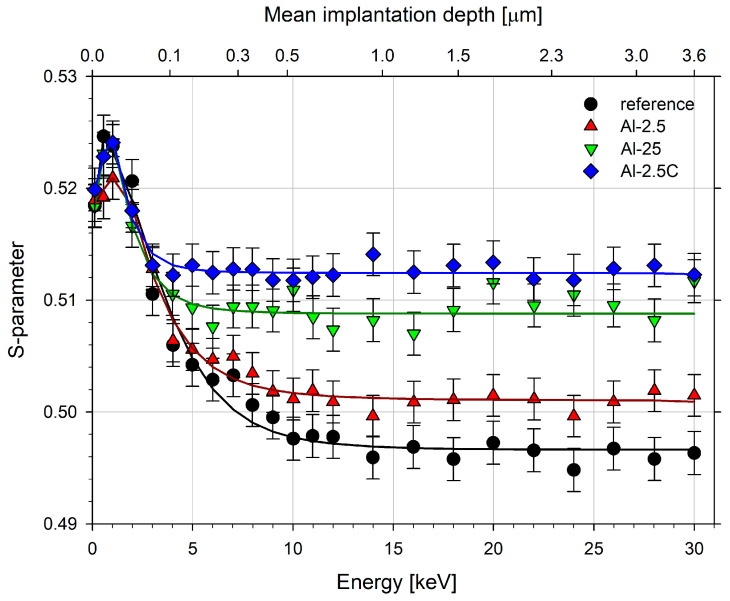
The dependencies of the *S* parameter on the incident positron energy for the extruded aluminum samples and the reference sample. Solid black lines represent the best fit using the VEPFIT code [82].

Figure 2 shows the dependencies of the *S* parameter on the positron implantation energy (bottom axis) and the mean implantation depth (top axis) for the extruded samples and the well-annealed reference one. The mean implantation depth $\bar{z}$ was calculated using the formula [81]:

(1) $$\bar{z}=\frac{AE^{n}}{\rho },$$

where *E* is the positron energy in keV, and *A* and *n* are the Makhovian function parameters.

The initial increase in the *S* parameter at the surface visible for all samples is caused by a thin surface oxide layer and an interface between this layer and the bulk metal [83,84]. For the positron energy higher than 0.1 keV, the *S* parameter decreases and finally reaches a constant value distinctive for each sample. Solid lines represent the best fits obtained using the VEPFIT code [82] with parameters *A* = 2.53 µg cm^−2^ keV^−*n*^, *n* = 1.748, *ρ* = 2.70 g/cm^3^ taken for aluminum from Ref. [85]. The thickness of the oxide layer obtained from VEPFIT was a few nanometers for all the studied samples. The values of the positron diffusion length and the *S* parameter for the bulk of the extruded aluminum samples and the reference one, *S*_bulk,_ are given in Table 3. The positron diffusion length for the reference sample, found to be close to 100 nm, is lower than the 150 nm estimated in the literature, e.g., Ref. [83]. This can be related to the lower purity of the commercial AA1050 aluminum samples or the presence of residual lattice defects. Nevertheless, a reduction in the positron diffusion length (*L*_+_) is observed in all extruded samples compared to the reference material. This reduction is relatively small for sample Al-2.5, while significantly larger for samples Al-2.5C and Al-25. Similarly, the increase in the bulk value of the *S*-parameter (*S*_bulk_) for Al-2.5 is much smaller than that for Al-2.5C and Al-25. These results indicate that the concentration of lattice defects introduced during RT extrusion with the reduction ratio (*R*) of 2.5 is lower than that produced by the extrusion of a cryo-cooled sample at a similar *R* or room temperature HE at *R* = 25. This further confirms the superior sensitivity of the positron annihilation technique compared to X-ray diffraction.

**Figure 3:**
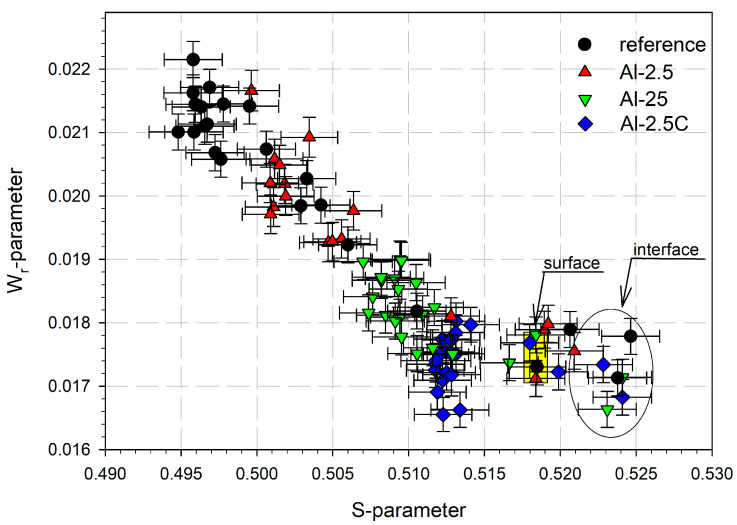
The dependencies of the *S* parameter as a function of the *W*_r_ parameter for the extruded aluminum samples and the reference sample.

The mutual dependence of the *W*_r_ and *S* parameters is shown in Figure 3. The so-called *S*-*W*_r_ plot can reveal information about the changes in the types of lattice defects in the sample. When only two positron states with a variable relative input contribute to annihilation, the *S*-*W*_r_ dependence is proportional, and the plot shows a straight-line segment. The endpoints of this segment correspond to the lattice defects or phases exhibiting specific values of the *S* and *W*_r_ parameters. It is visible in Figure 3 that the points originating from positron annihilation on the surface, in the oxide layer, or at the interface deviate from the straight line along which the majority of the experimental points are roughly arranged. The upper left end of this line corresponds to positron annihilation in the bulk of the reference sample. The lower right end corresponds to positron annihilation in the bulk of the Al-2.5C sample with the highest concentration of lattice defects. Therefore, it can be assumed that the lattice defects induced by HE are similar for the three samples studied, differing only in concentration, which is the highest for the Al-2.5C sample and the lowest for the Al-2.5 one.

Two lifetime components can be resolved in the PALS spectra obtained for the extruded samples, i.e., $\tau_{1}$ and $\tau_{2}$. Their values and intensity of the second component, *I*_2_, are gathered in Table 4 ($I_{1}+I_{2}=1$). The mean positron lifetime, $\bar{\tau }$, calculated as follows:

(2) $$\bar{\tau }=I_{1}\tau_{1}+I_{2}\tau_{2}$$

is also given in Table 4. The mean positron lifetime is a robust parameter that does not depend on the spectrum deconvolution procedure and allows for a more reliable assessment of the extent of changes in the lattice defect type and concentration connected to the changes in the microstructure.

The mean positron lifetime changes from 190 to 203 ps (Table 4). For the sample Al-2.5, it is lower than for the other two samples, which reflects the behavior of *S*_bulk_ obtained in the VEP beam measurements. This suggests a lower average density of crystal lattice defects, taking into account that these defects are similar in all samples. The longer lifetime, $\tau_{2}$, in the range 247–251 ps, comes from the annihilation of positrons trapped in defects. The shorter lifetime, $\tau_{1}$, has values close to 130 ps, which are much lower than the so-called bulk positron lifetime obtained for well annealed Al, i.e., 162 ps. It is well explained in accordance with the two-state positron trapping model or a positron diffusion trapping model [86,87]. In the case of a dislocation cell structure or small grains, if the cells/grains are small enough, positrons can diffuse to the cell walls/grain boundaries and be trapped in defects in those regions. Inside the cells/grains, positrons can annihilate from a free state in the undisturbed crystal lattice, where their lifetime is shorter, or become trapped in defects, if such are present inside cells/grains, contributing to the longer component. Then, the shorter lifetime component originates from positron annihilation in areas of low lattice defect concentration. Its presence indicates that, despite relatively high deformation, regions with a low concentration of the lattice defects remain.

It is known that the distribution of lattice defects induced by deformation is not homogeneous, resulting in a cellular dislocation substructure with low dislocation density in cell interiors and high dislocation density in cell walls and subgrain boundaries. The cell size decreases with deformation [88]. Dynamic recovery favors such a type of microstructure, which is more pronounced for deformation at relatively high homologous temperatures. This is consistent with the results of the TEM study of HE aluminum of commercial purity, performed by Pachla et al. [29]. Those authors demonstrated that the product extruded to a true strain of nearly 1, which is only slightly higher than that of the Al-2.5 and Al-2.5C samples, exhibited a microstructure consisting of subgrains within the micrometer-sized primary grains. The subgrain size of approximately 900 nm is typical for both cryo-cooled and RT extruded products in this case. Thus, the microstructure of both products (i.e., Al-2.5 and Al-2.5C) should be similar despite significantly different deformation temperatures. For the RT extruded material with a strain close to 3, Pachla et al. reported smaller subgrains (i.e., 600 nm). It should be noted, however, that the samples investigated by Pachla et al. were additionally cooled with water to limit static recovery after they left the die [29]. Therefore, the subgrain sizes of the samples investigated in the present paper may be slightly larger. Nevertheless, the currently studied samples do not differ significantly in terms of the hardness and yield strength from those quenched and studied in previous research [29]. Therefore, we do not expect a significant difference in the subgrain size of the material in the current study. Indeed, Styczyński et al. showed that the water cooling after extrusion did not affect the grain size, and the deformation-induced microstructure effect was limited mainly to a change in relative dislocation density inside the grains [89]. The positron lifetime in aluminum lattice defects has been extensively studied both experimentally and theoretically. Fluss et al. reported a positron lifetime of 244 ps for monovacancies in polycrystalline aluminum at 350 °C [90]. Theoretical calculations performed for Al by Häkkinen et al. indicated a positron lifetime of 252 ps for bulk monovacancies [91]. For divacancies, the theoretically calculated positron lifetime is larger and equal to 273 ps [92]. The positron lifetime for vacancies associated with dislocations, which have lower symmetry than thermally generated monovacancies, was experimentally determined to be equal to 220 ps for the Al single crystal [93,94]. Theoretical calculations gave the values of 224–225 ps [95]. However, Čížek et al. attributed a higher positron lifetime value of 243 ps to positrons trapped at vacancies associated with dislocations in high-purity polycrystalline aluminum rolled at 77 K [87].

The results obtained by us for the extruded samples are close to those obtained by Su et al. [87] and Cao et al. [96] for commercially pure Al grade AA1050 processed by equal-channel angular pressing (ECAP) at room and cryogenic temperatures, for which $\tau_{2}$ was in the range of 220–251 ps and about 241 ps, respectively. In addition to positron annihilation in vacancies associated with dislocations, Su et al. also considered the contribution to $\tau_{2}$ from positron annihilation in bulk monovacancies and divacancies to explain the longest lifetimes [87].

The highest mean positron lifetime and the shortest positron diffusion length obtained for the Al-2.5C sample indicate the highest average defect density. The clear difference between the Al-2.5C sample and Al-2.5 one is particularly noticeable, even though, according to Pachla et al., the difference in structure and grain size between cryo-cooled and RT extrusion at low strains is small due to comparable adiabatic effects [29]. The higher average defect density in the Al-2.5C sample may be related not only to the higher density of dislocations near the subgrain boundaries, but also to their presence in the subgrain interior.

As reported by Pachla et al., due to recovery, the material extruded at RT at higher strains exhibits grains with a dislocation-free interior and a higher density of dislocations at grain boundaries [29]. The mean positron lifetime and positron diffusion length for the samples Al-2.5C and Al-25 have similar values despite the differences in microstructure and grain size. However, a slightly higher average defect density is still found in the cryo-cooled sample extruded at a much lower true strain. Therefore, it can be concluded that microstructure refinement has a significant contribution to the strain hardening of hydroextruded aluminum. The difference in subgrain size between 600 and 900 nm explains the higher yield strength of the Al-25 sample than the Al-2.5 one [97]. The highest value of the cooled sample Al2.5C can be related to the higher dislocation density inside the subgrains, and therefore to increased dislocation hardening.

#### 3.2.2. Isochronal Annealing of the Extrusion Products

**Figure 4:**
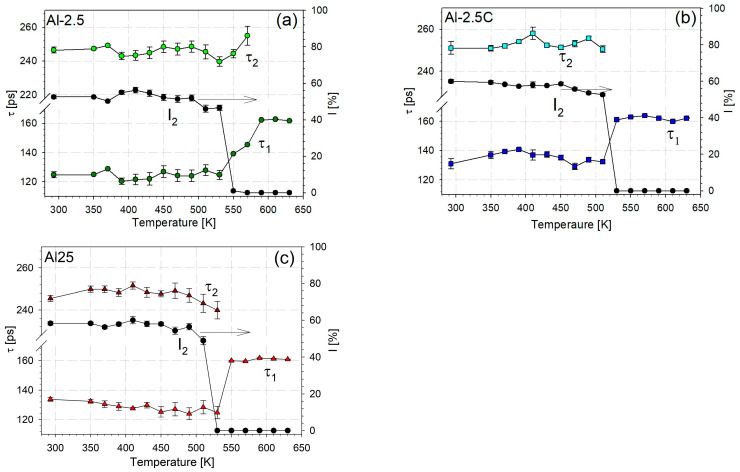
Dependencies of the positron lifetimes $\tau_{1}$, $\tau_{2}$ and intensity of the long lifetime I2 on the annealing temperature: (**a**) Al-2.5; (**b**) Al-2.5C; (**c**) Al-25.

Figure 4 shows the dependencies of two positron lifetime components and the intensity of the longer of them, *I*_2_, on the annealing temperature. In all cases, it can be seen that the values of $\tau_{1}$, $\tau_{2}$, and *I*_2_ do not change significantly up to a temperature of about 500 K. A steady, slight decrease in *I*_2_, indicating a slight reduction in defect concentration, precedes its rapid decline to virtually zero. This slight decrease can be attributed to static recovery of the material. In the case of the Al-2.5 sample, a clear but gradual decrease in *I*_2_, ending with the disappearance of this component, occurs in the temperature range of 530–590 K. This decrease in *I*_2_ is accompanied by an increase in *τ*_1_, which finally reaches the bulk value. For samples Al-2.5C and Al-25, *I*_2_ drops virtually to zero at temperatures of 530 and 550 K, respectively. Such behavior indicates that the recrystallization of the Al-2.5 sample occurs within the temperature range of 530–590 K, while for the remaining samples, it ends at temperatures of approximately 530 and 550 K. This relatively low difference corresponds to the variations in the deformed material microstructure, texture, and properties. As expected, the recrystallization temperature of the less deformed sample Al-2.5 is slightly higher than that of the other two.

**Figure 5:**
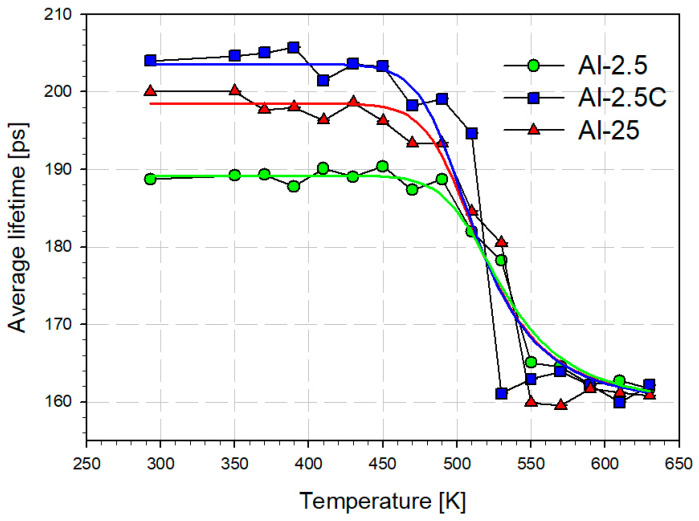
The mean lifetime vs. temperature annealing for AA1050 aluminum samples.

**Figure 6:**
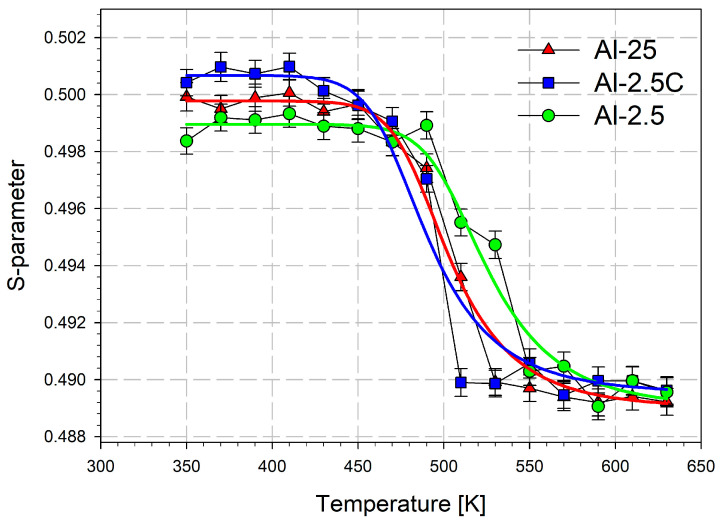
The *S* parameter vs. the annealing temperature.

The temperature dependencies of the mean positron lifetime $\bar{\tau }$ and the *S* parameter are shown in Figure 5 and Figure 6, respectively. For the samples Al-2.5C and Al-25 the slight decrease in $\bar{\tau }$ and the S-parameter caused by static recovery starts above 400–430 K, which agrees with observations of Pachla at all. [29]. It is least visible at about 450 K for the sample 2.5Al with the lowest initial defect concentration. The drop in the mean positron lifetime $\bar{\tau }$ starting at 450–470 K for the Al-2.5C and Al-25 samples and at 490–510 K for the Al-2.5 sample indicate the beginning of recrystallization, which ends earliest in the case of the Al-2.5C sample. This is visible both in the course of the $\bar{\tau }$ and *S* parameters (Figure 5 and Figure 6). Additionally, the range of changes in both parameters $\bar{\tau }$ and *S*), which can be associated with the extent of recrystallization, is smallest for Al-2.5C and largest for Al-2.5.

The temperature dependencies of the *S*-parameter or $\bar{\tau }$ resulting from recrystallization can be used in the diffusion trapping model to determine the activation energy of the grain boundaries migration during recrystallization [98]. The model assumes positron annihilation in the defect-free interior of the new grains. Positrons can also diffuse to the grain boundaries, where they become trapped at crystal lattice defects and then annihilate. The model takes into account grain growth kinetics induced by temperature. Thus, the increase in temperature causes grain boundary migration, which leads to an increase in the grain size reflected in the decreasing *S*-parameter or $\bar{\tau }$ values. However, the positron diffusion length of approximately 0.1 μm allows tracing for only the initial stage of recrystallization when the grain radii reach several micrometers. For larger, well-annealed grains, positron trapping and annihilation at grain boundary regions can be neglected. The solid curves in Figure 5 and Figure 6 are the results of fitting the model describing the grain boundary migration to the experimental points for the studied samples. The values of activation energy of the grain boundary migration, obtained for these two relationships, were used to calculate a weighted average taking into account their uncertainties. The results are gathered in Table 5. The obtained values are close to 1.5 eV and vary little from sample to sample. They are higher than 0.63 ± 0.1 eV determined for high-purity aluminum subjected to a rapid solidification process [99]. They are also larger than the activation energy for grain boundary diffusion in aluminum, which is 0.87 eV, as measured by TEM [88]. However, alloying elements in aluminum raise the activation energy for grain boundary migration. For example, 1 wt.% of Au increases it to 1.73 ± 0.40 eV [99]. Therefore, values near 1.5 eV may result from about 0.5% impurities (mainly Fe and Si) in the studied grade AA1050. Although the uncertainties in the energy values obtained from the fitting procedure are comparable to the differences between the samples, a pattern can be observed. Thus, the smallest activation energy of the grain boundary migration for cryo-cooled Al-2.5C coincides with this sample’s slightly lower recrystallization temperature. In turn, the highest energy is shown by the least deformed Al-2.5 sample.

## 4. Conclusions

1. Distinct axial textures were found both for the hydrostatically extruded and post-deformation annealed samples of aluminum grade AA1050. The relative contributions of the main components of the axial texture, i.e., <111> and <100>, depend on the HE conditions. An increase in the contribution of the <100> texture component for the cryo-cooled material can be related to the effect of the low temperature and high stress rate of deformation. Recrystallization causes an increase in the intensity of the <100> texture component, combined with a decrease in the intensity of the <111> one.
2. Shortening in the positron diffusion path, more significant for the cryo-cooled material at the true strain of 0.9 and that extruded at RT at the strain of 3.2, resulted from increased density of the lattice defects. This was also reflected in the bulk values of the *S* parameter and the mean positron lifetime.
3. Vacancies associated with dislocations are the main positron-trapping defects revealed by PALS. However, the contribution of monovacancies or even divacancies cannot be excluded.
4. The cryo-cooled material extruded at a true strain of 0.9, as well as the one extruded at RT at a true strain of 3.2, exhibited significantly higher mean lattice defect concentrations and considerably lower recrystallization temperatures compared to the sample extruded at RT at a true strain of 0.9, which had a less defective crystal structure.
5. The activation energy for grain boundary migration during the recrystallization of hydrostatically extruded AA1050 aluminum, as determined from the positron diffusion trapping model, is approximately 1.5 eV and showed only a slight dependence on the extrusion temperature and true strain.

## Figures and Tables

**Table 1:** Specification and parameters for AA1050 samples of the HE process.

Sample	ET	EP [MPa]	*R* = *A*_0_/*A*_f_	TS ln*R*	MES [1/s]	Hardness HV0.2	UTS R_m_ [MPa]	YS R_0.2_ [MPa]	Elongation at Break *ε* [%]
initial	-	-	-	-	-	29	76	51	36
Al-2.5	room	217	2.49	0.91	1.37	46	125	118	15.7
Al-2.5C	cryo-cooled	175	2.51	0.92	2.02	47	138	132	14.6
Al-25	room	180	25.3	3.23	5.55	49	129	121	16.4

ET—Extrusion temperature, EP—Extrusion pressure, *R*—Reduction ratio (*A*_0_—the cross-section surface area of the billet, *A*_f_—the cross-section surface area of the extruded product), TS—True strain, MES—Mean extrusion speed (value independent of the geometry of the system), UTS—Ultimate tensile strength, YS—Yield strength.

**Table 2:** XRD measurement results.

Sample	Al-2.5C		Al-2.5		Al-25	
	Extruded	Annealed	Extruded	Annealed	Extruded	Annealed
Lattice parameter [Å]	4.0504(2)	4.0499(4)	4.0504(2)	4.0502(2)	4.0510(3)	4.0503(2)
Crystallite size [Å]	371(36)	479(118)	382(28)	381(51)	381(61)	332(21)
Lattice strain [%]	−0.04(2)	−0.06(4)	−0.04(1)	−0.06(2)	−0.06(3)	−0.08(1)

**Table 3:** The values of the positron diffusion length *L*_+_ and *S*_bulk_ parameter for the extruded aluminum samples obtained from VEPFIT (solid lines in Figure 2).

Sample	*L_+_* [nm]	*S*_bulk_
reference	97(8)	0.4964(3)
Al-2.5	78(10)	0.5010(3)
Al-2.5C	16(5)	0.5124(2)
Al-25	22(2)	0.5092(2)

**Table 4:** Positron lifetimes and intensities.

Sample	$\tau_{1}$ [ps]	$\tau_{2}$ [ps]	$I_{2}$ [%]	$\bar{\tau }$ [ps]
Al-2.5	125 ± 2	247 ± 2	53 ± 1	190
Al-2.5C	131 ± 3	251 ± 3	60 ± 2	203
Al-25	133 ± 2	249 ± 2	58 ± 1	200

**Table 5:** The activation energy of the grain boundary migration, *Q*, was obtained from the diffusion trapping model [98].

Sample	*Q* [eV]
Al-2.5	1.51 ± 0.21
Al-2.5C	1.45 ± 0.36
Al-25	1.50 ± 0.25

## Data Availability

The data presented in this study are available on request from the corresponding author due to technical/time limitations.

## Abbreviations

The following abbreviations are used in this manuscript:

PALS	Positron Annihilation Lifetime Spectroscopy
DB	Doppler Broadening
CAGR	Compound Annual Growth Rate
HE	Hydrostatic Extrusion
TEM	Transmission Electron Microscopy
RT	Room Temperature
XRD	X-ray Diffraction
TREOR	Trial and Error
ODF	Orientation Distribution Function
ADC	Arbitrarily Defined Cell
VEP	Variable Energy Positron
YS	Yield Strength
UTS	Ultimate Tensile Strength
ET	Extrusion Temperature
EP	Extrusion Pressure
R	Cross-Section Area Reduction Ratio
TS	True Strain
MES	Mean Extrusion Speed
fcc	Face Centered Cubic
SFE	Stacking Fault Energy
ECAP	Equal-Channel Angular Pressing
